# Dosimetric Impact of Interfractional Variations in Prostate Cancer Radiotherapy—Implications for Imaging Frequency and Treatment Adaptation

**DOI:** 10.3389/fonc.2019.00940

**Published:** 2019-09-27

**Authors:** Tilman Bostel, Ilias Sachpazidis, Mona Splinter, Nina Bougatf, Tobias Fechter, Constantinos Zamboglou, Oliver Jäkel, Peter E. Huber, Dimos Baltas, Jürgen Debus, Nils H. Nicolay

**Affiliations:** ^1^Clinical Cooperation Unit “Radiation Oncology”, German Cancer Research Center, Heidelberg, Germany; ^2^Department of Radiation Oncology, Heidelberg University Hospital, Heidelberg, Germany; ^3^Department of Radiation Oncology, University Medical Center Mainz, Mainz, Germany; ^4^Department of Radiation Oncology, University of Freiburg Medical Center, Freiburg, Germany; ^5^German Cancer Consortium (DKTK), Partner Site Freiburg, German Cancer Research Center, Heidelberg, Germany; ^6^Heidelberg Institute of Radiation Oncology, National Center for Radiation Research in Oncology, Heidelberg, Germany; ^7^Medical Physics in Radiation Oncology, German Cancer Research Center, Heidelberg, Germany

**Keywords:** prostate cancer, image-guided radiotherapy, dosimetry, organs-at-risk, tumor control probability

## Abstract

**Background and purpose:** To analyze deviations of the applied from the planned doses on a voxel-by-voxel basis for definitive prostate cancer radiotherapy depending on anatomic variations and imaging frequency.

**Materials and methods:** Daily in-room CT imaging was performed in treatment position for 10 patients with prostate cancer undergoing intensity-modulated radiotherapy (340 fraction CTs). Applied fraction doses were recalculated on daily images, and voxel-wise dose accumulation was performed using a deformable registration algorithm. For weekly imaging, weekly position correction vectors were derived and used to rigidly register daily scans of that week to the planning CT scan prior to dose accumulation. Applied and prescribed doses were compared in dependence of the imaging frequency, and derived TCP and NTCP values were calculated.

**Results:** Daily CT-based repositioning resulted in non-significant deviations of all analyzed dose-volume, conformity and uniformity parameters to the CTV, bladder and rectum irrespective of anatomic changes. Derived average TCP values were comparable, and NTCP values for the applied doses to the bladder and rectum did not significantly deviate from the planned values. For weekly imaging, the applied D_2_ to the CTV, rectum and bladder significantly varied from the planned doses, and the CTV conformity index and D_98_ decreased. While TCP values were comparable, the NTCP for the bladder erroneously appeared reduced for weekly repositioning.

**Conclusions:** Based on daily diagnostic quality CT imaging and voxel-wise dose accumulation, we demonstrated for the first time that daily, but not weekly imaging resulted in only negligible deviations of the applied from the planned doses for prostate intensity-modulated radiotherapy. Therefore, weekly imaging may not be adequately reliable for adaptive treatment delivery techniques for prostate. This work will contribute to devising adaptive re-planning strategies for prostate radiotherapy.

## Introduction

Prostate cancer is among the most prevalent malignant diseases in men with between 100 and 170 newly diagnosed patients per 100,000 people annually (1). Radiotherapy is one of the mainstays of prostate cancer therapy, and several large analyses have suggested outcomes comparable to surgical tumor removal while resulting in favorable toxicities in the surrounding organs-at risk (2–4). The use of intensity-modulated radiotherapy (IMRT) has been shown to reduce late radiation-induced genitourinary and gastrointestinal toxicities, thereby enabling the safe application of higher treatment doses (5, 6). However, the advent of high-precision radiotherapy for prostate cancer has made the treatment application more susceptible to inaccuracies due to changes in the pelvic anatomy, and the occurrence of intra- and especially interfractional variations of the prostate and the surrounding organs-at risk have been well studied (7–9). Regular image guidance using cone-beam CT (CBCT) is set to reduce inaccuracies in dose application due to anatomic changes; nevertheless, the validity of the anatomic information is somewhat hampered by the weak soft tissue contrast of pelvic CBCT imaging and may require additional means of patient position control like implanted fiducials. The implications of interfractional variations on the applied radiotherapy doses are less well understood. Previous work using weekly CT scans and rigid registration demonstrated significant deviations from the prescribed dose for both IMRT and proton radiotherapy (10, 11). More recent work using elastic imaging registration reported no clinically relevant aberrations of the applied doses from the doses prescribed during a prospective study; however, this analysis relied on weekly imaging and CBCT-based dose accumulation (12).

Here, we quantified interfractional anatomic variations and calculated resulting deviations of the applied from the planned doses using daily planning CT scans performed for positional verification in treatment position as part of the daily radiotherapy algorithm. Additionally, the impact of the frequency of position control imaging on the applied radiation doses was studied. This work will contribute to devising adaptive re-planning strategies for prostate radiotherapy.

## Materials and Methods

### Patient Selection

Ten consecutive patients receiving definitive prostate radiotherapy at the German Cancer Research Center were included in this analysis. All patients presented with low or intermediate-risk prostate cancer with stage T1c to T2b disease, Gleason scores not exceeding 7 and PSA values ranging below 20 ng/ml (13). This study is in accordance with the Declaration of Helsinki (Seventh Revision, 2013) and was approved by the Independent Ethics Committee of the Medical Faculty of the University of Heidelberg, Germany (S-380/2017).

### Treatment Planning and Delivery

For all patients, the clinical target volume (CTV) comprised the prostate gland for low-risk tumors and additionally 10–15 mm of the proximal seminal vesicles for intermediate-risk cancers. A setup margin of 7 mm was added to the CTV to create a planning target volume (PTV). The prescribed dose was 76.50 Gy in 34 fractions of 2.25 Gy. Dose constraints to the organs-at risk (OAR) were based on the Quantitative Analyses of Normal Tissue Effects in the Clinic (14–16). Patients were immobilized with a ProStep™ pelvic and lower extremity support (Elekta, Stockholm, Sweden), and were instructed to present to daily treatment with an empty bowel and a comfortably filled bladder. Treatment plans were generated using the RayStation planning system (RaySearch Laboratories, Stockholm, Sweden), and step-and-shoot IMRT was applied using 9 co-planar fields on an Artiste linear accelerator (Siemens, Erlangen, Germany).

### Daily CT Imaging

Patients were positioned in treatment position on the treatment couch as described above, based on tattooed skin markers applied at the time of the planning CT and a calibrated 3D laser coordinate system. The treatment couch was then rotated into a CT scanner (Primatom; Siemens OCS, Malvern, USA) that was integrated into the linear accelerator setup and located at a 90° angle directly adjacent to the accelerator in the treatment room. Patients received daily diagnostic CT imaging in treatment position as position verification; the treatment couch was then re-rotated to the treatment gantry with no manipulation to the patient setup. The in-room CT scanner had been approved for treatment planning scans, and all scans were taken to the same specifications used for the individual planning examinations.

### Analysis of Variations and Dose Accumulation

Target volumes and OARs were outlined by a board-certified radiation oncologist both on the planning CT scans and the daily position verification CT scans according to current contouring guidelines (17, 18). Daily fraction doses were recalculated on the corresponding daily images, and resulting dose distributions were transferred onto the planning scans. Voxel-wise dose accumulation was carried out in RayStation using the software's deformable image registration module and compared to the planned dose distribution (19). Daily imaging was rigidly registered to the planning CT scans prior to dose re-calculation as done for routine patient repositioning. For simulated weekly imaging, position correction vectors were derived from the CT scans of days 1, 6, 11, 16, 21, 26, and 31 and used to rigidly register the five daily scans of each treatment week to the planning CT scan. Applied and planned 3D dose distributions were compared using dose-volume indices, including mean dose (D_mean_), doses at × % volume (D_x_) and volume at x Gy doses (V_x_). The conformity-related indices, conformity index (CI) and conformal index (COIN) for the total prescription dose, and the uniformity parameters gEUD and gEUD_2Gy_ were also considered for comparison purposes (see Annex I in Supplementary Material) (20, 21). In addition, the tumor control probability (TCP) and normal tissue complication probabilities (NTCP) for the relevant OARs and the complication-free tumor control probability (P_+_), were calculated and considered for the analysis (see Annex II in Supplementary Material).

Finally, the applied dose distributions were compared within the region receiving >10% of the maximum dose by 3D-gamma analysis to the clinical tolerance level of 3%/3 mm (22).

### Statistical Analysis

Statistical analysis of the differences in dose volume indices between planned and applied doses was performed by Wilcoxon signed-rank test with corresponding two-sided confidence intervals using in-house software developed in Python (https://www.python.org). An alpha level of 0.05 was used for all statistical tests.

## Results

### Anatomic Variations

**Figure 1:**
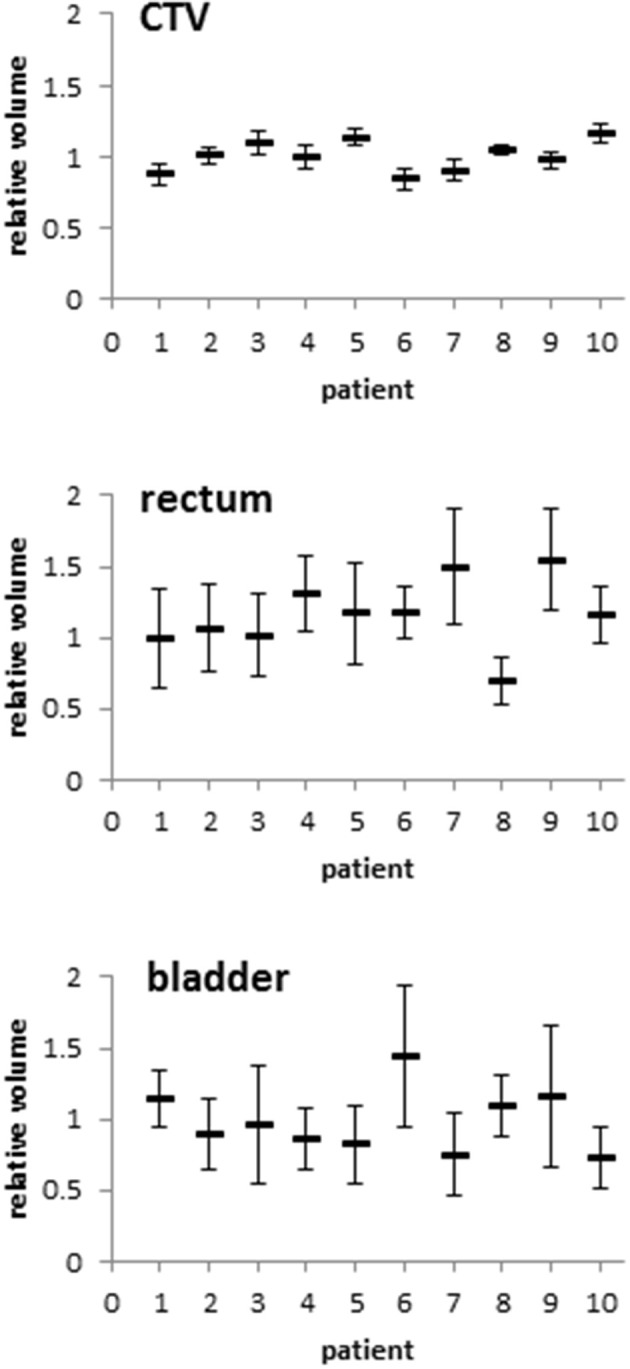
Relative volumes of the CTV, rectum and bladder of each patient during the course of radiotherapy compared to the planning CT-based volumes. Error bars represent standard deviation.

**Figure 2:**
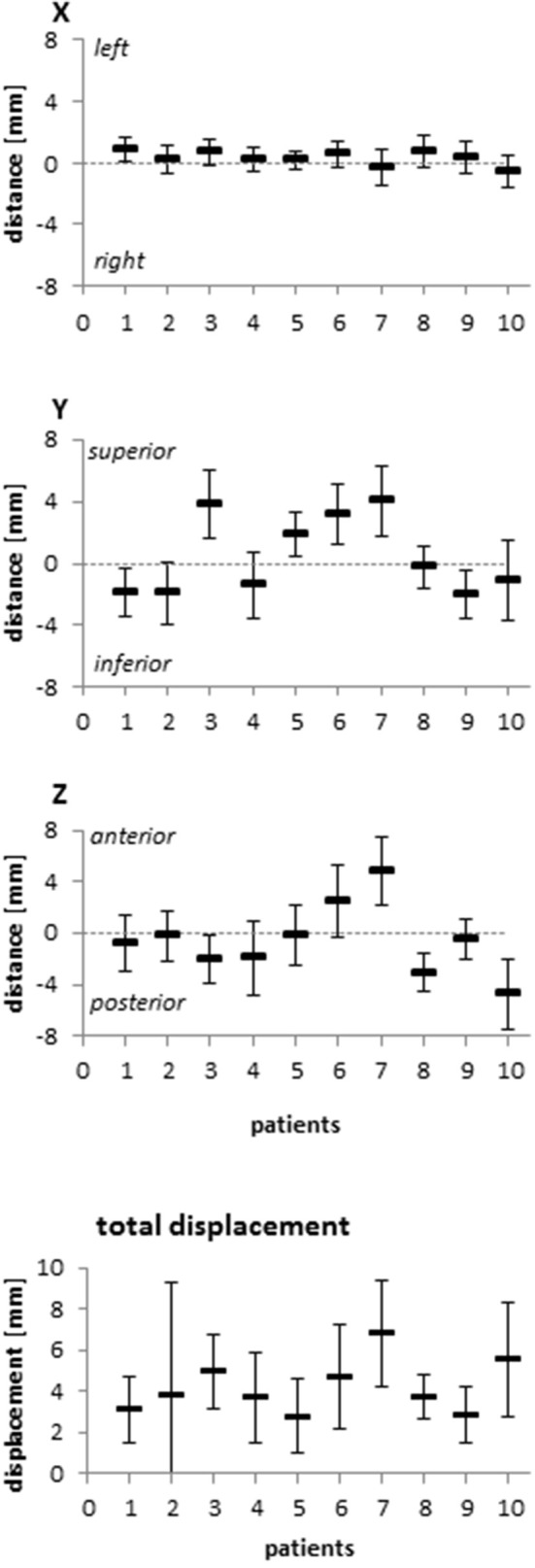
Relative deviation of the geometric center of the CTV between the planning CT and the fractional CTs in all three dimensions and resulting total displacement for each patient included in this analysis. Error bars represent standard deviation.

Patients exhibited distinct anatomic changes during the course of radiotherapy. While the pre-treatment CTVs ranged between 37.5 and 168.0 ml for analyzed patients, the volumes remained stable throughout therapy with a relative volume difference between the planning examination and the treatment scans of 0.3 ± 10.9% (Figure 1, Supplementary Figure 1). Rectal volumes were measured between 36.4 and 112.4 ml at treatment planning and increased by an average of 9.8 ± 32.3% during treatment (Supplementary Figure 1). As expected, the bladder volumes exhibited the biggest variability, ranging between 71.5 and 530.4 ml on the planning CT scan; however, the average difference to the bladder volumes during treatment was only 1.3 ± 22.2%. Regarding individual patients, considerable differences in rectal and bladder filling during treatment were observed. To assess potential implications of the described anatomic changes on the position of the CTV, its geometric center was compared between the planning scan and the daily scans after individual rigid pre-treatment realignments based on adjacent bony structures. The average lateral CTV displacement in the lateral X direction amounted to 0.3 ± 0.3 mm (fractional range: −0.2 to 0.9 mm), and there were only small effects observed for daily treatment fractions or individual patients (individual range −0.6 to 0.9 mm) (Figure 2, Supplementary Figure 2). Changes in the superior-inferior Y direction amounted to an average of 0.4 ± 0.7 mm (fractional range: −1.5 to 2.1 mm) with considerably more inter-individual variability (individual range −2.0 to 4.1 mm). Similarly, the anterior-posterior CTV displacement resulted in an average shift of −0.6 ± 0.7 mm (fractional range −1.6 to 1.5 mm) with strong differences for individual patients (individual range −4.7 to 4.9 mm). Due to the defined PTV margin of 7 mm, daily pre-treatment analysis of position verification CTs did not warrant treatment adaptation or re-planning for any patient based on interfractional variability.

### Influence of Anatomic Variations on Dose Distribution

**Figure 3:**
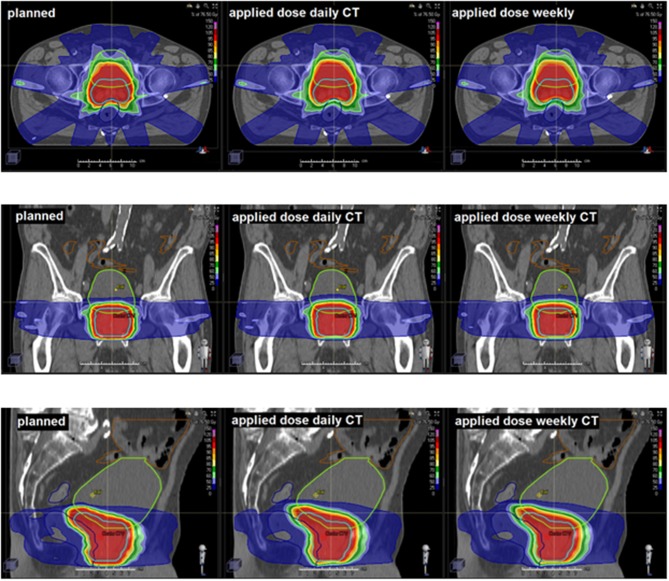
Representative CT slices demonstrating the planned and applied doses using daily or weekly CT-based repositioning.

**Figure 4:**
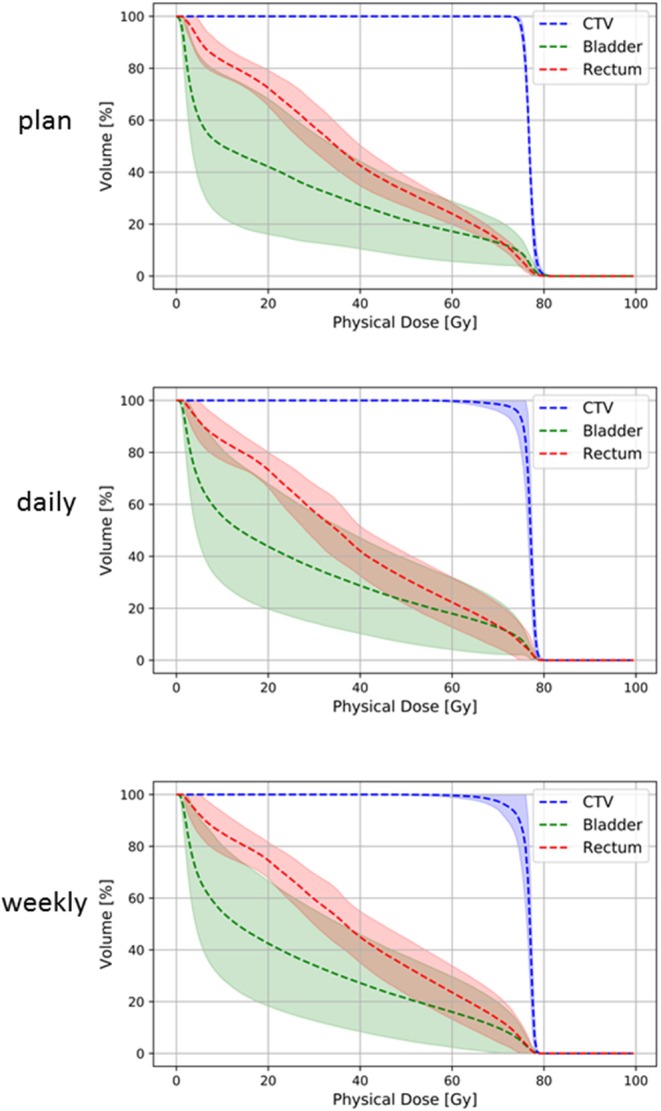
Summary dose-volume histograms for the CTV (blue line), rectum (red line) and bladder (green line) for treatment plans **(upper panel)** and accumulated doses after daily **(middle panel)** and weekly **(lower panel)** repositioning. Lighter-colored bands represent the 95% confidence interval of each dose-volume curve.

Applied doses were compared with the planned doses to the CTV, bladder and rectum (Figure 3). As expected from the volumetric analyses, the bladder demonstrated the highest variability between planned and applied doses, and the deviations for bladder and rectum were strongest in the high dose range (Figure 4). Table 1 summarizes the differences in the dose-volume indices between planned and accumulated doses. For the CTV, the strongest deviations were seen for D_98_ with an average reduction of 2.27 Gy for the accumulated dose; in contrast, the accumulated average D_mean_ and D_2_ deviated by only −0.08 and −0.40 Gy from the prescribed doses, respectively. No significant differences in the gEUD and gEUD_2Gy_, the conformity indices or the probability of complication-free tumor control (P_+_) could be observed between treatment plans and applied doses.

**Table 1:** Average and standard deviation of differences between applied and planned dose-volume indices for daily or weekly CT-based repositioning.

		**Daily imaging**	***P*-value**	**Weekly imaging**	***P*-value**
CTV	D98 (Gy)	−2.27 ± 5.05	0.54	−4.24 ± 6.00	0.19
	D50 (Gy)	0.13 ± 0.74	0.26	0.01 ± 0.75	0.36
	Dmean (Gy)	−0.08 ± 1.05	0.61	−0.47 ± 1.07	0.36
	D2 (Gy)	−0.40 ± 0.83	0.42	−0.83 ± 0.90	**0.04**
	V76.5 (%)	0.09 ± 0.27	0.22	0.05 ± 0.26	0.36
	EUD (Gy)	−0.39 ± 1.69	0.76	−1.32 ± 2.54	0.42
	gEUD (Gy)	−1.15 ± 3.77	0.76	−3.31 ± 6.56	0.36
	CI	0.16 ± 0.61	0.54	−0.35 ± 0.42	**0.03**
	COIN	0.06 ± 0.12	0.13	0.09 ± 0.14	0.13
	P+	0.00 ± 0.02	0.31	0.02 ± 0.04	0.19
Bladder	D50 (Gy)	2.78 ± 5.88	0.10	1.84 ± 5.67	0.08
	D70 (Gy)	1.55 ± 5.90	0.10	1.18 ± 5.91	0.10
	Dmean (Gy)	1.40 ± 4.60	0.19	0.15 ± 4.98	0.61
	D2 (Gy)	−2.34 ± 6.06	0.22	−4.55 ± 8.48	**0.02**
	V75 (%)	−0.01 ± 0.04	0.68	−0.04 ± 0.05	**0.01**
	V70 (%)	0.00 ± 0.05	1.00	−0.03 ± 0.06	0.19
	V55 (%)	0.01 ± 0.06	0.42	−0.01 ± 0.07	0.92
	V45 (%)	0.01 ± 0.06	0.48	0.00 ± 0.07	1.00
	EUD (Gy)	−0.94 ± 3.95	0.68	−3.43 ± 5.44	0.22
	gEUD (Gy)	−1.42 ± 4.76	0.68	−4.65 ± 6.59	0.08
Rectum	D50 (Gy)	0.41 ± 3.73	0.76	2.78 ± 4.10	0.07
	D70 (Gy)	0.38 ± 2.41	0.84	0.63 ± 3.55	0.48
	Dmean (Gy)	−0.12 ± 3.85	0.61	0.89 ± 3.58	0.42
	D2 (Gy)	−1.54 ± 2.91	0.08	−2.04 ± 2.77	**0.02**
	V75 (%)	0.01 ± 0.07	0.48	−0.01 ± 0.06	0.76
	V70 (%)	−0.01 ± 0.08	0.41	−0.01 ± 0.09	1.00
	V50 (%)	−0.01 ± 0.09	0.31	0.01 ± 0.09	0.84
	V40 (%)	−0.01 ± 0.08	0.68	0.02 ± 0.07	0.22
	EUD (Gy)	−1.32 ± 5.28	0.31	−1.18 ± 5.08	0.54
	gEUD (Gy)	−1.75 ± 6.46	0.22	−1.87 ± 6.28	0.48

*Negative values represent decreases in accumulated doses. Bold values indicate statistically significant*.

**Figure 5:**
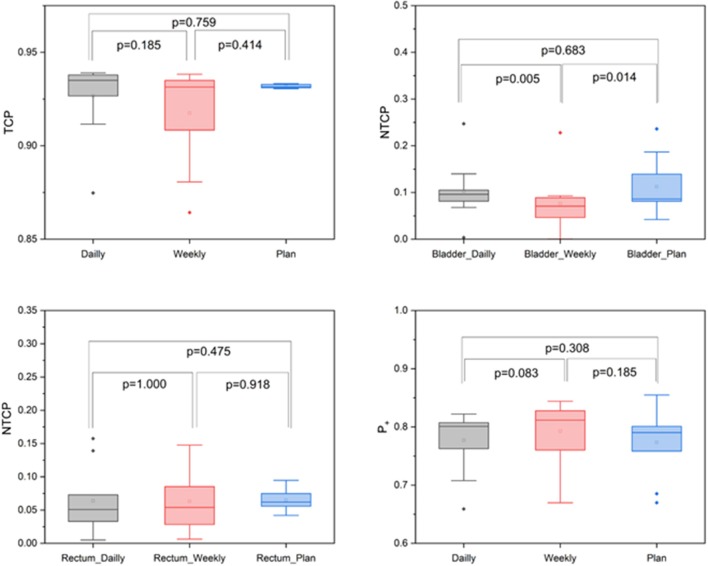
Box-plot diagrams for TCP, bladder and rectal NTCP and P_+_ values derived from the planned and accumulated doses.

The accumulated average D_mean_ to the rectum was only 0.14 ± 3.73 Gy higher than planned, and all dose-volume indices did not show significant deviations from the treatment plan. Considering the considerable volume changes for the bladder, the applied D_mean_ was on average 2.78 ± 5.88 Gy higher than initially planned. However, all dosimetric values for the bladder also varied only insignificantly, and the mean dose-volume parameters were highly comparable between planned and accumulated doses. Similarly, EUD-based comparison including gEUD and gEUD_2Gy_ did not show any significant differences for the rectum or bladder. The median prostate TCP values were 93.16% (min. 93.05%, max. 93.33%) for the planned dose and 93.5% (min. 87.48%, max. 93.9%) for the applied dose with no significant deviation (*p* = 0.759) (Figure 5). Similarly, NTCP values resulting from the accumulated and the planned doses were highly comparable, and no significant differences were found for the bladder (*p* = 0.683) or the rectum (*p* = 0.475). P_+_ values were also comparable between planned and applied doses (*p* = 0.308).

### Dosimetric Impact of Daily vs. Weekly Positioning Imaging

**Figure 6:**
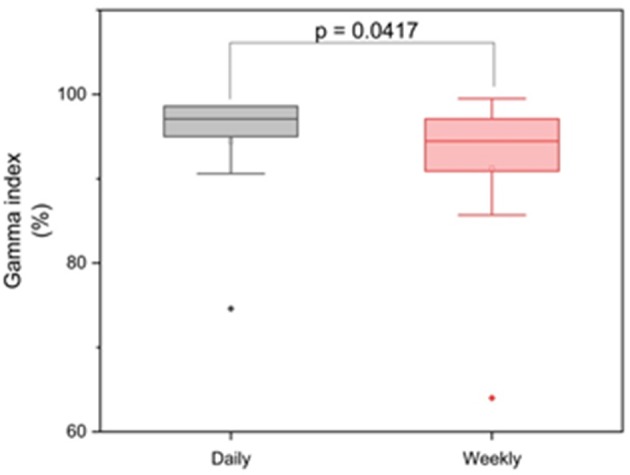
Box-plot diagram for the gamma analyses regarding daily and weekly imaging.

To evaluate the dosimetric impact of daily vs. weekly position verification CT, correction vectors from the first positioning scan of each treatment block (fractions 1, 6, 11, 16, 21, 26, and 31) were used to rigidly register all consecutive weekly CTs to the planning scan, and applied doses were accumulated and compared to accumulated doses resulting from daily CT-based repositioning. The largest deviations from the planned dose-volume indices were observed in the high dose range, with the D_2_ significantly lower for the CTV (−0.83 ± 0.90 Gy; *p* = 0.040), the bladder (−4.55 ± 8.48 Gy; *p* = 0.020) and the rectum (−2.04 ± 2.77 Gy; *p* = 0.020) (Figure 4, Table 1). Weekly repositioning also resulted in a significant decrease of the CTV conformity index compared to the planning data (−0.35 ± 0.42; *p* = 0.030) and a decrease in the D_98_ (−4.24 ± 6.00 Gy; *p* = 0.190), although the latter value did not reach statistical significance. There was also a trend toward increased median doses to the rectum (2.78 ± 4.10 Gy; *p* = 0.070) for weekly repositioning. TCP and P_+_ values were not significantly different between applied and planned doses irrespective of the repositioning frequency. Similarly, NTCP values for the rectum did not significantly deviate for the weekly or daily repositioning, while NTCP for the bladder was found lower when repositioning was only performed once a week (Figure 5). The gamma passing rate to the clinical tolerance level of 3%/3 mm was 3.2% lower for the weekly position verification imaging than that for the daily imaging (*p* = 0.042) (Figure 6).

## Discussion

Intra- and inter-fractional anatomic variations have been a concern for prostate cancer radiotherapy, and the potential dosimetric consequences of these variations have been subject to intense research; however, studies have been hampered by the quality and frequency of positional imaging and the availability of sufficient image registration tools, and to the best of our knowledge, to date, no publications are available using CT-based daily voxel-wise dosimetric comparisons for intensity-modulated prostate radiotherapy. This dataset provides for the first time a comprehensive analysis of the dosimetric impact of interfractional variability using daily in-room planning-quality CT imaging, enabling adequate voxel-wise dose accumulation for the target volume and organs-at-risk over the course of intensity-modulated radiotherapy for each fraction and each patient. Our data demonstrated that daily CT-based repositioning resulted in only non-significant deviations of all analyzed dose-volume parameters to the CTV, bladder and rectum from the planned doses irrespective of anatomic changes in the bladder and rectal filling and position. In contrast, reliance on weekly position-verification imaging resulted in deviations especially for the high doses and significantly reduced dose conformity to the target volume. To the best of our knowledge, this is the first dataset utilizing daily diagnostic CT imaging and a deformable registration strategy to enable voxel-wise dose tracking for prostate cancer patients undergoing IMRT.

In the past, several strategies for the accumulation of the applied dose in prostate cancer radiotherapy have been reported for available CBCTs in order to overcome the limited usability of these low-quality images, including portal dose measurements, using enhanced CBCT with additional filters or rigid registration strategies with limited dose mapping potential (7, 12, 23, 24). To date, voxel-wise dosimetric analyses based on daily CTs are only available for proton radiotherapy; this may be due to a broader availability of diagnostic scanners in proton treatment facilities and the necessity of higher-quality imaging given the increased anatomy-dependent range uncertainties of protons (25). A previous report analyzing proton treatment data demonstrated significant lower dose in the PTV in 15 out of 225 analyzed treatment fractions, mostly caused by alterations in the rectal volume (26). In a second publication analyzing 10 patients undergoing proton radiotherapy, the V_72.67_ was above 95% for 90.4–98.7% of treatment fractions depending on the matching strategy (25). To our knowledge, similar high-quality data are not available for IMRT.

Considering the variable pelvic anatomy and the influence of bladder and rectal filling on the localization of the prostate, the ideal frequency of positional verification imaging has been subject to some debate, and several publications have analyzed dosimetric and clinical implications of daily vs. weekly imaging. A previous analysis based on CBCTs from 20 patients demonstrated improved target coverage and reductions of the V_50_, V_65_, and D_mean_ to the rectum with daily imaging, although no voxel-wise dose accumulation could be performed due to limitations in image quality and registration (27). Similar improvements of target coverage by daily imaging have been reported in other publications, and it has been suggested that PTV margins should be adjusted according to the imaging frequency (28). In our dataset, daily image-guided repositioning resulted in better target volume conformity and target volume coverage and a trend toward increased rectal doses. However, the impact of these dosimetric improvements for patients remains to be fully elucidated (29). To date, at least two prospective trials have investigated the clinical implications of daily CBCT for prostate cancer patients. In a French randomized study analyzing 470 men, daily imaging resulted in improved biochemical and clinical progression-free survival and reduced late rectal toxicity, while having an adverse correlation with patients' overall survival (30). A second randomized trial did not demonstrate any advantage regarding toxicities or patients' quality of life from daily CBCT and reduced PTV margins compared to weekly portal imaging (31).

The additional use of implanted fiducial markers for prostate radiotherapy may also impact potential deviations of the applied from the prescribed doses; however, it has been suggested that there are only minor deviations in the dose distribution between patients with and without markers if 3D imaging is performed daily (7). Using implanted markers, kV X-ray imaging is commonly used to replace daily CT imaging or to supplement weekly 3D imaging schedules, but does not show bladder and rectal anatomy and hence does not allow an assessment of dosimetric effects on these organs.

Future developments may help to decrease dosimetric deviations of prostate radiation treatments from treatment plans by optimizing repositioning: the advent of MR-linac and the opportunity for real-time MR-guided prostate tracking before and during each treatment fraction may enable adjustments of patient positions based on superior soft tissue contrast and may also help to minimize not only interfractional but also intrafractional motion (32, 33). Additionally, repositioning strategies have been proposed that utilize accumulated dosimetric information over the course of treatment for dose-guided patient repositioning (34). However, in our dataset, dosimetric deviations in all tested dose-volume parameters from the treatment plan were non-significant when daily 3D imaging was performed, making potential further benefits for the treated patients debatable.

Despite the high imaging quality and the comprehensive dosimetric information, there are limitations to our analysis. The number of patients included in this analysis was relatively small due to logistical reasons associated with the complex workflow, and the patient number may affect the statistical power. Additionally, dose deviations may not only depend on interfractional setup errors, but also on intrafractional prostate motion which could not be taken into account for our data. Additionally, all patients included in our analysis were coached about bladder and rectal filling and also received feedback based on their imaging results on all days the bladder or rectal filling deviated considerably from the planning CT. The resulting relative consistency in bladder and rectal anatomy in all analyzed patients may be a key reason for the moderate dosimetric effects of our simulated weekly repositioning analysis, and patients may in reality exhibit considerably higher dose deviations in case of weekly imaging. Simulating weekly imaging by utilizing every fifth CT scan (weekly treatment block) was based on routine clinical imaging schedules for prostate cancer patients; however, it may potentially introduce a bias as this methodology only provides an anatomic snap-shot that may not be representative for other simulated non-daily imaging schedules.

The relevance of our findings for high-risk or locally advanced prostate cancers and prostatic bed irradiation will require further investigations, as different treatment margins for these scenarios will likely affect dose-volume parameters.

Nevertheless, our dataset provides a novel in-depth and voxel-wise analysis of the dosimetric effects of interfractional variations in prostate cancer radiotherapy based on a comprehensive dataset of daily high-quality CTs that may have implications for the frequency of verification imaging and may help to guide strategies for adaptive radiotherapy planning.

## Conclusion

Despite considerable variations in the position and volume of bladder and rectum, daily in-room CT-based rigid patient repositioning resulted in only negligible deviations of applied doses from planned doses for prostate radiotherapy. In contrast, voxel-wise dose accumulation analyses demonstrated significant divergences in the high dose range and a reduction of dose conformity to the target volume in case of weekly CT-based position verification. This indicates for the first time on a voxel-based level that a reduced imaging frequency may not be adequately reliable for adaptive treatment delivery techniques for intensity-modulated prostate radiotherapy. This work will contribute to devising adaptive re-planning strategies for prostate radiotherapy.

## Data Availability Statement

The datasets generated for this study will not be made publicly available. These are patient-individual data that are protected by German laws and cannot be distributed without explicit written consent by every included patient.

## Ethics Statement

This study was carried out in accordance with the recommendations of name of guidelines, name of committee with written informed consent from all subjects. All subjects gave written informed consent in accordance with the Declaration of Helsinki. The protocol was approved by the approved by the Independent Ethics Committee of the Medical Faculty of the University of Heidelberg, Germany (S-380/2017).

## Author Contributions

TB, PH, JD, and NN planned and carried out treatment. TB, IS, MS, TF, CZ, NB, OJ, DB, and NN analyzed data. NN wrote the manuscript. IS, PH, and DB helped with writing the manuscript. JD helped with data discussion.

### Conflict of Interest

The authors declare that the research was conducted in the absence of any commercial or financial relationships that could be construed as a potential conflict of interest.

## Supplementary Material

Supplementary Figure 1 Mean relative volumes of the CTV, rectum and bladder for each treatment fraction compared to the planning CT-based volumes. Error bars represent standard deviation.

Supplementary Figure 2 Relative deviation of the geometric center of the CTV between the planning CT and fractional CTs in X, Y, and Z direction **(A)** and resulting total displacement **(B)** for each treatment fraction. Error bars represent standard deviation.
