# Supplementary figures and images for: Dosimetric Impact of Interfractional Variations in Prostate Cancer Radiotherapy—Implications for Imaging Frequency and Treatment Adaptation
Source: Front Oncol. 2019 Sep 27;9:940. doi: 10.3389/fonc.2019.00940 (PMC6776888; doi:10.3389/fonc.2019.00940)

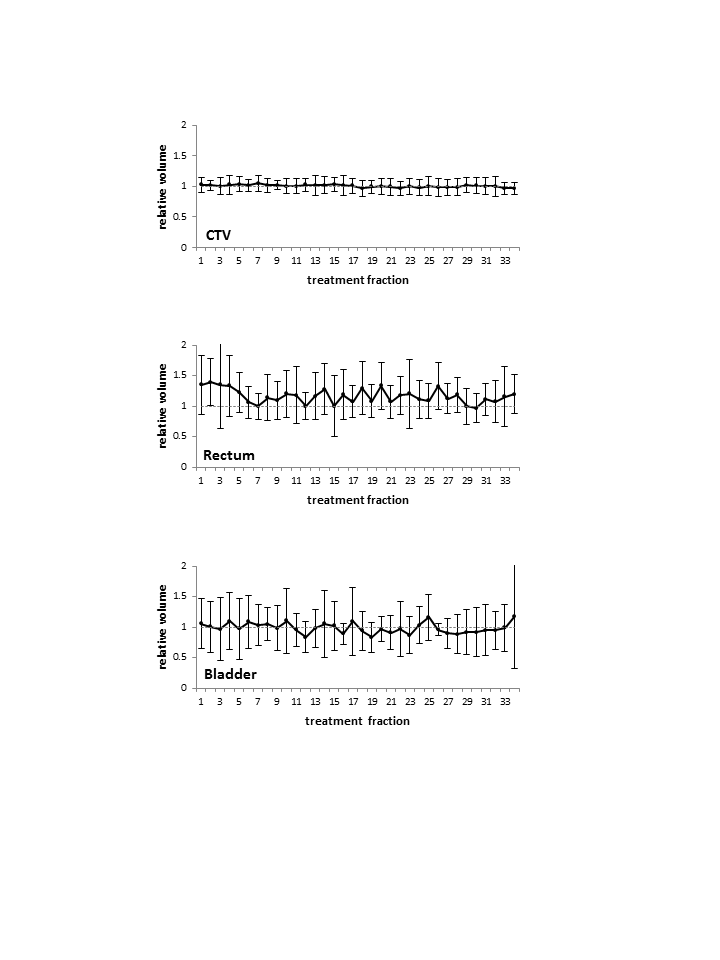

Supplement: Supplementary Figure 1 — Mean relative volumes of the CTV, rectum and bladder for each treatment fraction compared to the planning CT-based volumes. Error bars represent standard deviation. [file Image_1.TIF]

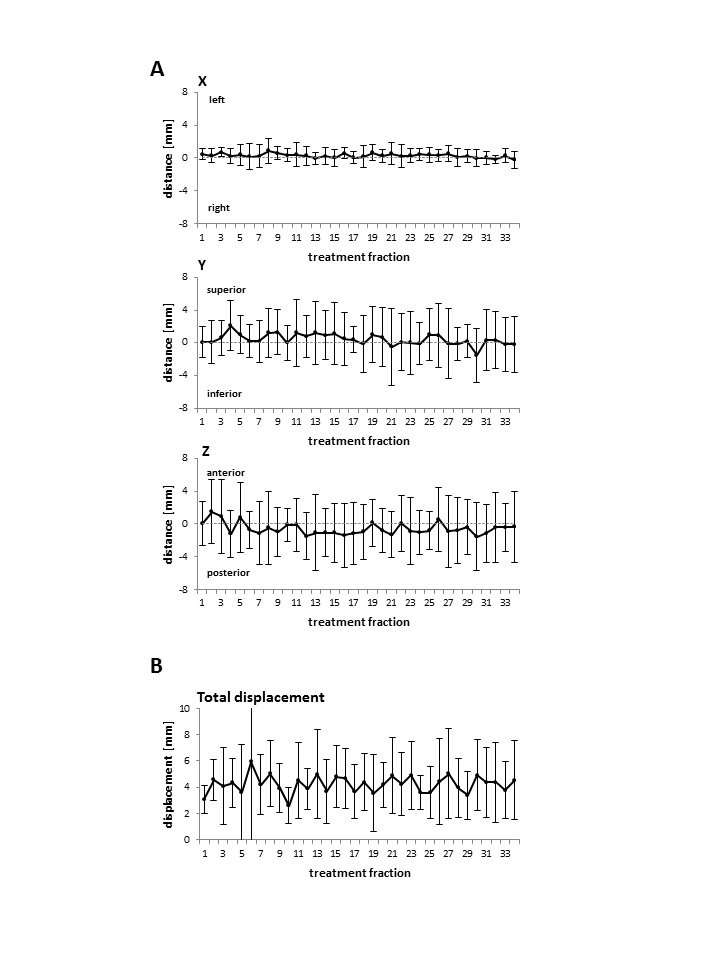

Supplement: Supplementary Figure 2 — Relative deviation of the geometric center of the CTV between the planning CT and fractional CTs in X, Y, and Z direction (A) and resulting total displacement (B) for each treatment fraction. Error bars represent standard deviation. [file Image_2.TIF]
