# Supplementary material for: Dosimetric Impact of Interfractional Variations in Prostate Cancer Radiotherapy—Implications for Imaging Frequency and Treatment Adaptation
Source: Front Oncol. 2019 Sep 27;9:940. doi: 10.3389/fonc.2019.00940 (PMC6776888; doi:10.3389/fonc.2019.00940)
Supplement: Supplementary file 3 [file Data_Sheet_1.docx]

**Annexes**

***Annex I***

### *The generalized equivalent uniform dose gEUD*

The generalized equivalent uniform dose (*gEUD*) is given for any VOI type (targets and OARs) by the following expression[34]:

$$gEUD=\left( \frac{1}{N}\sum_{i=1}^{N} D_{i}^{\alpha} \right)^{\frac{1}{\alpha}}$$

 (A1)

*N* is the number of dose sample points in the VOI and *D_i_* is the dose at the *i*^th^ calculation point. Generally *gEUD* is calculated based on the differential DVH of the corresponding VOI and in such a case *N* is the total number of DVH bins.

*gEUD* is based on the physical volumetric dose distribution. To account for the effects of different fractionation at different sampling points within the VOI, the *gEUD_2Gy_* quantity is also used. *gEUD_2Gy_* uses the 2Gy per fraction equieffective volumetric dose distribution as given below:

$${gEUD}_{2Gy}=\left( \frac{1}{N}\sum_{i=1}^{N} {EQD2}_{i}^{\alpha} \right)^{\frac{1}{\alpha}}$$

 (A2)

*EQD2_i_* is the equieffective dose at 2Gy per fraction of a total dose *D_i_* delivered at *d_i_* dose per fraction as this is calculated by:

$${EQD2}_{i}= \frac{D_{i}(1+ \frac{d_{i}}{a/\beta})}{1+ \frac{2 Gy}{a/\beta}}$$

 (A3)

The parameter *α* is the tumor-(*α*<0) or normal tissue-(*α>*0) specific parameter that describes the dose-volume effect of the anatomic structure of interest.

***Annex II***

The response probability of the tumor (TCP) according to the linear Poisson model [35, 36] is:

$$TCP= TCP\left( \left\{ D \right\},V \right)= \prod_{i=1}^{M} \left[ e^{\left( -e^{e \gamma-\left( \frac{{EQD2}_{i}}{D_{50}} \right).\left( e \gamma-\ln\ln2 \right)} \right)} \right]^{\Delta v_{i}}$$

 (A4)

*EQD2_i_*is calculated according to Eq. (A3).

*TCP({D},V)* is the probability of eradicating the tumor of volume *V* irradiated with a dose distribution *{D}*.

*D50* is the equieffective dose at 2Gy per fraction, *EQD2*, resulting to 50% response probability, and *γ* is the maximum value of the normalized dose-response gradient. M is the total number of bins of the DVH, *D_i_* and *d_i_* are the corresponding total dose and dose per fraction for the *i*^th^ bin. *Δv_i_* is the volume corresponding to the *i*^th^ dose bin and V the total volume of CTV.

The response probability *NTCP* for an OAR of volume *V* irradiated with a dose distribution *{D}* is calculated using the relative seriality model[35, 36]**:**

$$NTCP=P\left( \left\{ D \right\},V \right)=\left[ 1-\prod_{i=1}^{M} {(1-{P(D_{i})}^{s})}^{\frac{V_{i}}{V}} \right]^{\frac{1}{s}}$$

 (A5)

with *P(D_i_)* the response probability for the OAR having the reference volume and being irradiated to dose *D_i_*:

$$P(D_{i})=e^{-(\frac{{EQD2}_{i}}{D50})(e\gamma-lnln2)}$$

(A6)

Where *D50* is the equieffective dose *EQD2* resulting to 50% complication probability and *γ* is the maximum value of the normalized dose-response gradient.

*V_i_/V* is the volume fraction being irradiated to dose *D_i_,* and *s* is the parameter which expresses the degree of seriality. Values of *s* close to zero indicate nearly parallel structured organs, where increasing *s* values characterise increasing seriality.

The overall probability of injury *P_I_* is given by:

$$P_{I}=1- \prod_{i=1}^{N_{OAR}} (1-{NTCP}_{i})$$

 (A7)

With *NTCP_i_* being the complication probability for the *i*^th^ OAR and *N_OAR_* is the total number of OARs under consideration, here *N_OAR_* = 2 (bladder and rectum). *TCP* and the *NTCPs* are calculated from the corresponding DVHs.

The uncomplicated tumour control probability *P*_+_ has additionally been considered for the comparison of the different plans. *P*_+_ is based on the tumour control probability (*TCP*) for the CTV (prostate) and the overall probability of injury *P_I_* and provides a robust method of evaluating and comparing complex 3-dimensional dose distributions. *P*_+_ is approximated by the difference of the beneficial probability *P*_B_ and the overall probability of injury *P_I_*:

$$P_{+}=P_{B}-P_{B\cap I} \approx P_{B}- P_{I}$$

 (A8)

The term $P_{B\cap I}$ describes the probability of achieving tumor control together with a severe damage to normal tissue or OARs.The beneficial probability *P*_B_ in our case comprises of one target volume (CTV) and is therefore equal to the calculated *TCP*, Eq. (A4).

***Annex III***

Parameter values for the TCP and NTCP (relative seriality model) and gEUD-based calculations are depicted in table A1. The column *s* denotes the relative seriality index. *D50* is defined as equieffective dose value *EQD2*.

| **VOI** | ***D50* (Gy)** | ***γ*** | ***s*** | ***α/β* (Gy)** | **clinical endpoint** | ***α***  **volume effect**  **for *gEUD[37]*** |
| --- | --- | --- | --- | --- | --- | --- |
| Prostate | 58.3 | 1.99 | --- | 1.93 | 5 years relapse-free survival [38](*) | -10.0 |
| Bladder | 80.0 | 2.59 | 1.30 | 3.0 | Symptomatic bladder contracture and volume loss[39, 40] | 6.0 |
| Rectum | 80.0 | 1.79 | 0.75 | 3.0 | Symptomatic proctitis, necrosis, fistula, stenosis[39, 40] | 6.0 |

(*)The dose response model of [38] for the low-risk patient group has been fitted to the *TCP*-model of equation A4.
